# BEREAVED PARENTS OF THE HEALTH AND RETIREMENT STUDY

**DOI:** 10.1093/geroni/igad104.2499

**Published:** 2023-12-21

**Authors:** Stephanie Rubinstein, Gillian Fennell, Elizabeth Zelinski

**Affiliations:** University of Southern California, Los Angeles, California, United States; University of Southern California, Los Angeles, California, United States; University of Southern California, Los Angeles, California, United States

## Abstract

The loss of a child—no matter their age—is often regarded as one the most tragic forms of loss. Here, we sought to identify sociodemographic, psychosocial, and health profiles of older adults who have experienced offspring death in the Health and Retirement Study (HRS). Our sample was composed of 2,035 respondents from the 2018 wave of HRS who experienced (n =381) or did not experience (n = 1,654) the loss of a child at any point in their lifetime. In an adjusted logistic regression, we found that both women (OR=1.53, [1.13, 2.09]) and non-Hispanic Black respondents (OR = 1.85; [1.31, 2.61]) had significantly higher odds of experiencing the death of a child. Education was also significantly associated with offspring death: relative to individuals with less than a high school education, high school and college-educated respondents had a 34% and 64% lower likelihood of reporting offspring death, respectively (OR=0.66, [.47, .91]; OR=0.36, [.25, .51]). Lastly, every one unit increase in cognition was associated with a 3% lower likelihood of experiencing the offspring death (OR=0.97, [.94, .99]). Interestingly, bereaved parents were no more likely to experience depression nor report multimorbidity than individuals who had not grieved the loss of a child. These findings highlight a grim picture of premature and untimely death among Black Americans, the power of education, and potential psychological and physiological resilience among this older sub-population of bereaved parents.

